# SPICE Implementation of the Dynamic Memdiode Model for Bipolar Resistive Switching Devices

**DOI:** 10.3390/mi13020330

**Published:** 2022-02-19

**Authors:** Fernando Leonel Aguirre, Jordi Suñé, Enrique Miranda

**Affiliations:** Departament d’Enginyeria Electrònica, Universitat Autònoma de Barcelona (UAB), 08193 Barcelona, Spain; jordi.sune@uab.cat

**Keywords:** memristor, resistive switching, memory, memdiode

## Abstract

This paper reports the fundamentals and the SPICE implementation of the Dynamic Memdiode Model (DMM) for the conduction characteristics of bipolar-type resistive switching (RS) devices. Following Prof. Chua’s memristive devices theory, the memdiode model comprises two equations, one for the electron transport based on a heuristic extension of the quantum point-contact model for filamentary conduction in thin dielectrics and a second equation for the internal memory state related to the reversible displacement of atomic species within the oxide film. The DMM represents a breakthrough with respect to the previous Quasi-static Memdiode Model (QMM) since it describes the memory state of the device as a balance equation incorporating both the snapback and snapforward effects, features of utmost importance for the accurate and realistic simulation of the RS phenomenon. The DMM allows simple setting of the initial memory condition as well as decoupled modeling of the set and reset transitions. The model equations are implemented in the LTSpice simulator using an equivalent circuital approach with behavioral components and sources. The practical details of the model implementation and its modes of use are also discussed.

## 1. Introduction

**Figure 1:**
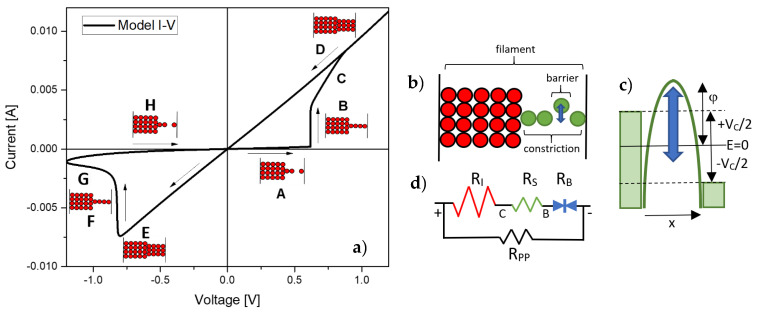
(**a**) Hysteretic behavior of the filamentary-type I-V characteristic. Filament stages: (A) barrier-controlled, high resistance state (HRS), (B) completion, (C) expansion, (D,E) low resistance state (LRS), (F) partial dissolution, (G) rupture and (H) HRS (**b**) Schematic of the filamentary structure: fixed (red) and dynamic (green, blue) sections. (**c**) Schematic of the tunneling barrier/gap. λ is the barrier height. (**d**) Representation of the QPC model using an equivalent circuit approach.

Selecting a suitable generic model for an electron device is far from being simple and straightforward. The model should be able to cover not only the basic and common features of the system under study but must also have the necessary capacity of adaptation for incorporating its distinctive behavior. For circuit simulation-oriented models, this adaptation capability must be achieved by means of a few numbers of simple and robust equations driven by a reduced set of parameters, if possible, with physical origin, if not, with some degree of electrical meaning. This is the signature of a compact behavioral approach, in which the central objective is not to outperform the microscopic level models (for instance the kinetic Monte-Carlo and Finite Element models such as those reported in [1,2,3,4]) in terms of accuracy and fidelity to the device physics, but matching observations and simulations as close as possible. Clearly, accurate representation of the electron transport across the investigated device under arbitrary input signals is two-fold: first, it encourages the design and assessment of more complex circuits and systems, and second, it allows to identify and establish links among the elementary modeling pieces that lead to the variety of observed behaviors (conduction characteristics). Since the first practical description of a memristive device by HP in 2008 [5], a number of compact models for the current-voltage (*I-V*) curves of resistive switching (RS) devices has been proposed [6]. Because of the hysteretic nature of the phenomenon (see Figure 1a), the electron transport model involves complementary information about the previous history of the device. Here is where Prof. Chua’s theory of memristive devices comes into play [7]. According to this theory, a physical or electrical variable expressed as a first order differential equation controls the current flow through the structure. Based on this framework, a plethora of memristor models have been proposed, such as the general phenomenological models (Yakopcic [8], TEAM [9], VTEAM [10], Eshraghian [11], etc.). Despite having a proven capability to successfully fit experimental data, these models rely on various internal equations or artificial window functions (commonly used for modelling the SET/RESET transitions) that can seriously affect the model’s convergence [12,13]. The reader can find further details for each model in the corresponding paper or in review papers [11,13,14,15,16,17,18].

Many of these models almost exclusively focus on the popular quasi-static, pinched *I-V* loop, ignoring the associated time-related dependencies. However, the latter has special relevance when considering real case application scenarios such as those described in [19], where the programming and reading of the device is made in terms of voltage pulses of varying frequency/duty cycle. In this regard, we aim to report the fundamentals and the SPICE implementation of a revised Dynamic Memdiode Model (DMM) for bipolar RS devices capable of incorporating the time-related dependencies, as well as the guidelines for its usage. Since this new version incorporates a dynamic balance equation for the memory state and a higher level of details in terms of modelling accuracy, we consider that it is a breakthrough with respect to the previous models proposed by our group: the Quasi-static Memdiode Model (QMM) [20,21,22] and a first version of the DMM [19]. The first one relies on the double-diode circuit controlled by the Krasnosel’skii-Pokrovskii hysteresis operator [23]. The main advantage of that simplified approach is the elimination of the time integration step in favor of the use of the so-called hysteron or memory map (i.e., the low-voltage conductance exhibited by the device as a function of the applied signal). However, even though a time module can be added to the base model [24], this is in essence a quasi-static approach in the sense that the memory state of the device does not change unless a hard threshold condition is met. The QMM can be used for arbitrary input signals, but the memory state is unable to evolve in the subthreshold regime. Seeking to overcome this limitation, we introduced the original DMM in [19].

In this work, we presented a revised version of the DMM. Compared to the previous version, the current one incorporates modifications capable of representing a variety of details that could not be modeled by its predecessor. These are: (*i*) the simulation of the electroforming event, which implies a first cycle different from its subsequent stationary cycles, (*ii*) the modelling of the snapback (SB) effect, experimentally observed in memristors during the SET event, (*iii*) the modelling of the snapforward (SF) event (low current tail) during the RESET event, and (*iv*) the de-coupling of the SET and RESET equations, which simplifies the fitting. The paper is organized as follows: Section 2 discusses the physical background of the model for both the transport and memory equations, as well as for the resulting switching dynamics. The dependences of the model with the fitting parameters are evaluated in Section 3, focusing on the memory equation, the switching dynamics, and the particular case of Complementary Resistive Switching (CRS) devices. Then, Section 4 presents an overview of the DMM capability for reproducing different sets of experimental data, from the quasi-static *I-V* loops as well as from the potentiation and depression dynamics for neuromorphic applications. Finally, Section 5 reports the conclusions of this work.

## 2. Dynamic Memdiode Model (DMM)

In this Section, the two equations that define the DMM are presented and discussed. They are: (*i*) the current-voltage (*I-V*) relationship, and (*ii*) the memory state equation (*λ-t*). The very basic idea behind the DMM is that the current flows through a kind of filamentary structure embedded in the oxide layer in which some of its atomic constituents can reversibly move in and out according to the forces exerted by the external field. This changing configuration alters in turn the overall transmission properties of the structure, leading the system to a hysteretic behavior. Since this is at the end a behavioral approach, the model can be appropriately modified so as to cover non-filamentary-type conduction as well.

### 2.1. Current-Voltage Characteristic

According to the quantum point-contact (QPC) model [25,26,27,28], the current that flows through a single nanosized filamentary structure (see Figure 1b) can be calculated using the finite-bias Landauer formula for a monomode conductor [29]:

(1) $$I\left(V_{C}\right)=\frac{2e}{h}\int_{-\infty }^{+\infty }T\left(E\right)\left[f\left(E-\frac{eV_{C}}{2}\right)-f\left(E+\frac{eV_{C}}{2}\right)\right]dE$$

where $V_{C}=V-IR_{I}$ is the potential drop across the constriction, *V* the applied voltage, $R_{I}$ the internal/external series resistance (permanent section of the filament/wire resistance), *E* the energy, *f* the Fermi-Dirac function, and *T* the transmission coefficient for the confinement barrier. *e* and *h* are the electron charge and the Planck constant, respectively. Equation (1) can be extended to the case of asymmetric potential drops at the two ends of the constriction using a coefficient different from ½ for the energy window. Assuming an inverted parabolic potential barrier for the constriction’s bottleneck (scatterer), *T(E)* is expressed as:

(2) $$T\left(E\right)=\frac{1}{1+e^{-\theta \;\left(E-\varphi \right)}}$$

where θ is the barrier shape factor and φ the barrier height (see Figure 1c). In the pure tunneling regime (φ≫E) and zero-temperature limit, Equations (1) and (2) yield [30]:

(3) $$I\left(V_{C}\right)\approx \frac{4e}{\theta \;h}e^{-\theta \varphi }sinh\left(\frac{\theta eV_{C}}{2}\right)$$

**Figure 2:**
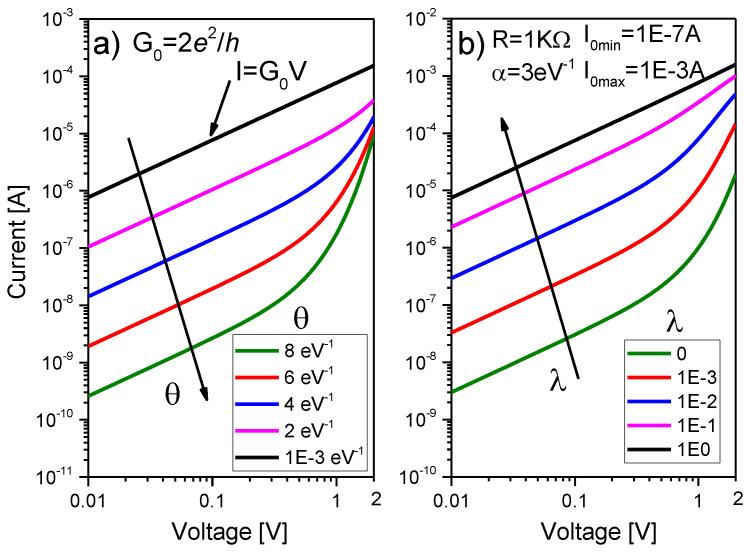
(**a**) Single filament conduction characteristic and effect of the parameter θ. (**b**) Multiple filament conduction characteristic and effect of the parameter λ.

Notice that, if the barrier width collapses (θ→0) because of the completion of the filament, Equation (3) results in the standard Landauer formula $I=G_{0}V_{C}$, where $G_{0}=2e^{2}/h$ is the quantum conductance unit. The effect of θ on the *I-V* curve is illustrated in Figure 2a. As θ increases, the current decreases, and the *I-V* changes its dependence from linear to linear-exponential. Combining Equation (3) in the linear regime ($eV_{c}\ll 2/\theta$) with Equation (2), we obtain:

(4) $$V_{C}\sim \frac{1}{G_{0}}e^{\theta \varphi }I=\frac{1}{G_{0}}\left(\frac{1-T\left(E=0\right)}{T\left(E=0\right)}\right)I$$

Since in a mesoscopic system, the constriction resistance $R_{C}$ can be regarded as the sum of the contact resistance $R_{L}$ and the scatterer resistance $R_{B}$ as [29]:

(5) $$R_{C}=\frac{1}{G_{0}T}=\frac{1}{G_{0}}+\frac{1}{G_{0}}\;\left(\frac{1-T}{T}\right)=R_{L}+R_{B}$$

we can associate Equation (4) with $R_{B}$. However, it is clear from Equation (5) that not only the confinement barrier contributes to the constriction resistance but also the way the constriction is attached to the charge reservoirs (or thermalizing region) through $R_{L}$. This is a well-known result [29] and is the consequence of the funneling effect of the electron wave function caused by the mismatch in the number of available energy states when passing from the reservoir to the constriction and vice versa.

Since, in general, for a wider constriction formed by a bunch of conducting channels neither the number *N* of elemental filamentary structures involved is known nor their specific barrier parameters $\theta_{i}$ and $\varphi_{i}$ can be accessed individually [31], we extrapolate Equation (3) to that case using the heuristic approximation:

(6) $$I\left(V_{C}\right)=I_{0}sinh\left[\alpha \left(V_{C}-R_{S}I\right)\right]$$

which has the same functional asymptotes as the original Equation (3) for large $I_{0}$ values $\left(R_{S}I\approx V_{C}\right)$ and low applied voltages $\left(R_{S}I\ll V_{C}\right)$ (see Figure 2b). While $R_{S}$ in Equation (6) accounts for the contact resistance, the hyperbolic sine function expresses the barrier resistance as illustrated in Figure 1d. $R_{PP}$ in the same figure deals with the device resistance before the forming event. A central difference between $R_{S}$ and $R_{I}$, is that $R_{S}$ will be allowed to change (if necessary) according to the memory state of the device (movement of ions/vacancies). Notice also that Equation (6) does not correspond strictly to *N* times the current flowing through a single filament, otherwise a parallel shift of Equation (3) towards higher current values would be obtained [32]. In addition, Equation (6) complies with the pinched condition I(V=0)=0 and, because of its heuristic nature, overcomes the physical limitation on the voltage drop $eV_{C}/2\leq \varphi$ imposed by Equation (3). Physically, the moving species in Figure 1b represent the hopping of ions/vacancies induced by the external applied field. As schematically illustrated in Figure 1c, the opening (RESET) or closing (SET) of the atom chain raises or lowers the top of the confinement barrier for the electron flow [33,34]. According to this picture, ballistic transport would not be required along the whole filament structure but just at the narrowest section of the constriction. For a complete discussion about conductance quantization effects in RS devices, see Refs. [35,36,37,38,39,40,41,42,43].

From the electrical viewpoint, Equation (6) can be envisaged as two opposite biased diodes in parallel with a single series resistance [6] (see Figure 1d). Since these are not real diodes, inverse saturation currents are disregarded. This was the approach followed in our previous works and justifies the origin of the name memdiode [23], i.e., a diode with memory. The main point is that Equation (6), as shown in Figure 2b, self-rectifies the *I-V* curve as the device switches from HRS (linear-exponential) to LRS (linear), which is in agreement with many experimental observations. Equation (6) is implemented in LTSpice XVII from Linear Technologies using two resistors ($R_{S}$ and $R_{I}$) in series with a behavioral voltage-controlled hyperbolic sine current generator with the amplitude factor given by the expression:

(7) $$I_{0}\left(\lambda \right)=\left(I_{on}-I_{off}\right)\lambda +I_{off}$$

where 0≤λ≤1 is the memory state variable, and $I_{off}$, $I_{on}$ are calibration parameters (OFF and ON currents). λ = 0 and λ = 1 correspond to HRS and LRS, respectively. The linear relationship between $I_{0}$ and λ is a key feature of the model and likely reflects the connection between the memory state and the density of conducting sites in the filamentary structure [44,45]. The effect of λ on the *I-V* curve is illustrated in Figure 2b. As λ increases, the *I-V* curve becomes more linear as expected for a fully formed conducting channel. Notice that, at low biases, the HRS *I-V* is in the linear tunneling regime (which must not be confused with ohmic conduction). Importantly, $I_{off}$ and $I_{on}$ control the barrier resistance and do not refer directly to the minimum and maximum currents that flow through the device. This will be ultimately determined by the whole system’s dynamics (hysteresis effect and series resistances). For the sake of completeness, α and $R_{S}$ in Equation (6) receive a similar treatment in the LTSpice script as that given to $I_{0}\left(\lambda \right)$. Both parameters can be swept from a minimum (OFF) to a maximum (ON) if required. If not, α and $R_{S}$ remain fixed. In the following Section, the memory state equation and its circuital implementation are discussed.

### 2.2. Memory State Equation

**Figure 3:**
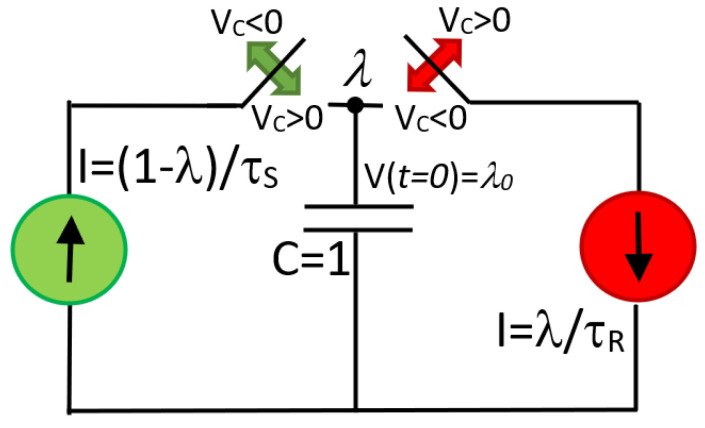
Equivalent circuit model for the memory state equation using two behavioral current sources and one capacitor. The switching $\tau_{S}$ and $\tau_{R}$ can be regarded as variable resistances. The state of the switches depends on the sign of the applied voltage. The memory state *λ* is the voltage across the capacitor. *λ_0_* is the initial memory state.

As reported in [46], a very convenient differential equation for the memory state variable λ that complies with a number of experimental observations in memristive structures is:

(8) $$\frac{d\lambda }{dt}=\frac{1-\lambda }{\tau_{S}\left(\lambda ,V_{C}\right)}-\frac{\lambda }{\tau_{R}\left(\lambda ,V_{C}\right)}$$

where $\tau_{S,R}$ are characteristic times associated with the SET (*V >* 0) and RESET (*V <* 0) transitions, i.e., with the ionic/defect movement within the dielectric film in one or the opposite direction. Equation (8) can be simply regarded as the normalized version of a birth-death process for a two-state system with transition rates $\tau_{S}^{-1}$ and $\tau_{R}^{-1}$:

(9) $$X\underset{\tau_{R}^{-1}}{\overset{\tau_{S}^{-1}}{⇄}}Y$$

in which there are $n_{1}$ particles in the state *X* and $n_{2}$ particles in the state Y, with $n_{1}+n_{2}=N$ the total number of particles. This comes to represent for example the REDOX process in VCMs [47]. Notice that $\tau_{S,R}$ in Equation (8) are expressed as a function of *V_C_* and λ. In our case, where SET and RESET only occur for biases with opposite signs, Equation (8) can be treated as two separate differential equations, one for *V >* 0 and one for *V <* 0. This is not mandatory but simplifies the model calibration since the SET and RESET processes are completely disentangled. Under this consideration, Equation (8) can be represented by the equivalent circuit schematically depicted in Figure 3. λ corresponds to the voltage drop across the capacitor *C =* 1 F. Notice that the memory state behavior during the RS cycle is nothing but the alternate action of two current sources that charge and discharge a capacitor. The green and red arrows in Figure 3 indicate the position of the switches as a function of the sign of *V_C_*. In practice, the switches are modeled by a behavioral *if* statement in the LTSpice script (Algorithm 1). The initial condition for the memory state is introduced through the initial voltage drop across the capacitor as *V*(*t* = 0) = *λ*_0_. In the LTSpice script, *λ* is represented by the capital letter H (for hysteron). In fact, *λ* = V(H) is the voltage at the H node. For the sake of completeness, and to help the reader in the use of the DMM, Supplementary Figures S1–S3 illustrates three simulation exercises in LTSpice obtained with the script reported in Algorithm 1.
**Algorithm 1:** Memdiode script for LTSpice XVII. + and − are the device terminals. H is the memory state output. The colors indicate the different sections: parameter values, memory equation, I-V characteristic, and auxiliary functions.**1****.subckt memdiode + − H****2***created by E.Miranda, F. Aguirre and J.Suñé, revised January 2022**3***.params***4**+ H0 = 0 ri = 50 RPP = 1E10**5**+ etas = 50 vs = 1.4**6**+ etar = 100 vr = −0.4**7**+ ion = 1E-2 aon = 2 ron = 10**8**+ ioff = 1E-7 aoff = 2 roff = 10**9**+ vt = 0.4 isb = 2E-4 gam = 1; isb = 1/gam = 0 no SB/SF**10***Memory Equation**11**BI 0 H I = if(V(+,-)> = 0, (1-V(H))/TS(V(C,-)),-V(H)/TR(V(C,-)))**12**CH H 0 1 ic = {H0}**13***I-V**14**RI + C {ri}**15**RS C B R = K(ron,roff)**16**BF B - I = K(ion,ioff)*sinh(K(aon,aoff)*V(B,-)) **17**RB + - {RPP}**18***Auxiliary functions**19**.func K(on,off) = off+(on-off)*limit(0,1,V(H))**20**.func TS(x) = exp(-etas*(x- if(I(BF)>isb,vt,vs)))**21**.func TR(x) = exp(etar* if(gam = = 0,1,pow(limit(0,1,V(H)),gam))*(x-vr))**22****.ends**

Both in the SET and RESET regions, the corresponding characteristic switching times can depend implicitly or explicitly on *λ*. This property is used to include the so-called snapback (positive bias) and snapforward (negative bias) effects in the RS *I-V* loop. These effects are typically present in VCM-based structures [48]. In this work, we introduce the memory state *λ* in the characteristic times as:

(10) $$\tau_{S}\left(\lambda ,V_{C}\right)=e^{-\eta_{S}\left(V_{C}-V_{S}\left(\lambda \right)\right)}$$

and

(11) $$\tau_{R}\left(\lambda ,V_{C}\right)=e^{\eta_{R}\lambda^{\gamma }\left(V_{C}-V_{S}\right)}$$

where $\eta_{S,R}$ and $V_{S,R}$ are the transition rates ($\eta_{S}$, $\eta_{R}>0$) and the reference switching voltages ($V_{S}>0$, $V_{R}<0$), respectively. γ≥0 is referred to as the SF coefficient. The exponential dependences of Equations (10) and (11) on *V_C_* are a consequence of the ions/vacancies dynamics associated with the hopping mechanism [49,50]. Deviations from these exponential laws in the low and high voltage regions have also been reported but are disregarded in this work [51].

If for any reason, the SB and SF effects do not need to be considered, taking *V_S_(λ) = V_S_* a constant reference SET voltage and $\lambda^{\gamma }=1$ in Equations (10) and (11), respectively, the switching dynamics becomes exclusively voltage-controlled, as originally assumed in [46]. This behavior is typical of ECM cells in which abrupt RESET transitions are observed [47]. Under these latter conditions, Equation (8) has an analytic solution both for the constant and ramped voltage input signal cases. In any other case, because of the mathematical complexity involved, Equation (8) must be numerically solved with the help of a differential equation solver (in our case the circuit simulator itself). In the following Section, the practical implementation and the consequences of the above-mentioned effects on the *I-V* curve are discussed.

## 3. Simulation Results and Discussion

In order to test the ability of the proposed model to deal with realistic simulations, a number of evaluation criteria must be adopted and assessed. In this work, we basically consider Linn’s criteria [52] to which we add some very specific features not included in the referred work. These criteria are: (*i*) capability of the compact model to reproduce the RS *I-V* characteristics including the SB and SF effects, (*ii*) realistic switching dynamics for the SET and RESET transitions including the ability of the model to deal with arbitrary input signals (continuous and discontinuous), and (*iii*) multi-device connectivity in the form of Complementary Resistive Switching (CRS). These three major issues are discussed in detail next. Before entering the discussion, it is worth mentioning that because of the complexity of the numerical problem involved, caution should be exercised with the selection of the model parameter values, as it happens with any other model. Although the DMM is robust enough, a certain combination of parameters could lead to fatal errors of convergence or to extremely long simulation times. Sometimes the numerical problems disappear by simply changing the maximum simulation timestep (shorter or longer) or numerical integration method used (trapezoidal, modified trap, gear). Depending on the required accuracy, simplifying the model equations by eliminating unnecessary details (SB, SF, resistances, limiting functions, etc.) is also a good strategy to follow. The roles played by parameters $I_{0}$, α, and $R_{S}$ in the HRS and LRS *I-V* curves are not analyzed here since they were discussed elsewhere in connection with the QMM [24]. Notice that the QMM can always be used as the starting point of any simulation exercise. The main difference between both models resides in how the SET and RESET transitions are modeled. While QMM requires a threshold voltage/current to induce the memory state evolution, DMM does not. The *I-V* expression in Equation (6) is common to both models.

### 3.1. Memory State Equation

**Figure 4:**
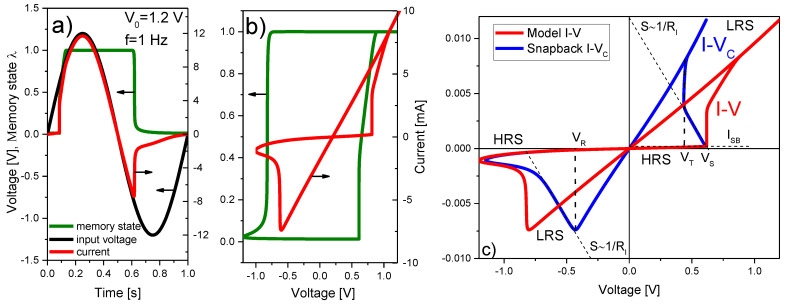
(**a**) Simulation example for the current and memory state as a function of time of a sinusoidal signal. (**b**) Evolution of the memory state (hysteron) and current as a function of the applied voltage. (**c**) Original *I-V* curve (red line) and its snapback correction *I-V_C_* (blue line). *V_T_* is the transition voltage, *V_R_* the reset voltage, *V_S_* the set voltage, *R_I_* the internal series resistance, and *I_SB_* the snapback triggering current.

To begin with, Figure 4 illustrates typical *I-V* and *λ-V* loops obtained using Equations (6) and (8). The LTSpice script and model parameters for this particular exercise can be found in Algorithm 1. While in Figure 4a, the sinusoidal input voltage, the memory state, and the current flowing through the structure are plotted as a function of time, Figure 4b illustrates the current evolution and the memory map (hysteron) of the device as a function of the applied voltage [53]. More in detail, the SB effect is recognized by the sudden current increase in the SET region (red line in Figure 4c) caused by the reduction of the constriction resistance that occurs when the tunneling gap or confinement barrier vanishes (the CF is completely formed). During this phase the current also grows as a function of *V_C_* but following approximately the load line of the circuit (slope~1/$R_{I}$), and next at an almost constant voltage called the transition voltage *V_T_* (blue line in Figure 4c) [48]. This second phase corresponds to the accumulation of ions/defects in the constriction (or alternatively to its lateral expansion) with the consequent progressive resistance reduction. This behavior has been reported many times in the literature [54] but has received scarce attention in the compact simulation field. *V_T_* is the minimum voltage required to activate the ions/vacancies movement and its value seems to be not only a characteristic parameter of each material but also a function of measurement variables such as the voltage ramp rate or signal frequency [55].

The SB effect is incorporated into the model equations by modifying the SET reference voltage *V_S_ (>V_T_)* in (10) according to the rule:

(12) $$V_{S}\left(I\right)=\{\begin{array}{cc} V_{T} & I\geq I_{SB}\\ V_{S} & I<I_{SB} \end{array}$$

where *I* is the current flowing through the device. Equation (12) is a switching rule based on the current value, but other rules related to the applied voltage, dissipated power, or memory state, are also admissible [56]. *I_SB_* is a threshold current for the SB effect. Equation (12) is written as an *if* statement for the SET voltage in the LTSpice script and expresses a collapse of the nominal SET voltage *V_S_* to a lower value *V_T_* after reaching the threshold condition *I_SB_*. This event generates a sudden current increase compatible with the voltage drop along the load line of the circuit. It is worth mentioning that the SB effect is not always observable in practice since its detection depends on a number of factors linked to the specific features of the device under test and to the measurement conditions (current magnitude, current compliance, etc.). When combined with other parameters (*I_off_* and *R_PP_*), *V_S_* can also be used to represent the forming step (see the Supplementary Material). This may require code edition for a specific conduction mechanism (Schottky, Fowler-Nordheim, etc.) in the fresh device [57].

For the opposite polarity (*V <* 0), after the SF event (current decrease following the circuit load line with slope~1/$R_{I}$), the main difference appears at the low current region, once the filament is almost dissolved. In this case, since λ approaches zero as the current drops, the factor $\lambda^{\gamma }$ gains weight in Equation (11), reducing the RESET characteristic time. The result is remarkable since the current deviates from the load line generating a lobe. In other words, as the current decreases, larger voltages are required to deplete the constriction from conducting atomic species up to the point in which the initial gap or tunneling barrier is completely restored. The referred protuberance is clearly visible in many VCM-type devices but is rarely observed in ECM-type structures, which exhibit more abrupt transitions [58,59]. Although *V_R_* is considered an independent model parameter in the LTSpice script, in general *V_R_ = −V_T_* is found, which is consistent with a field-induced activation of the SET/RESET processes in bipolar RS devices.

**Figure 5:**
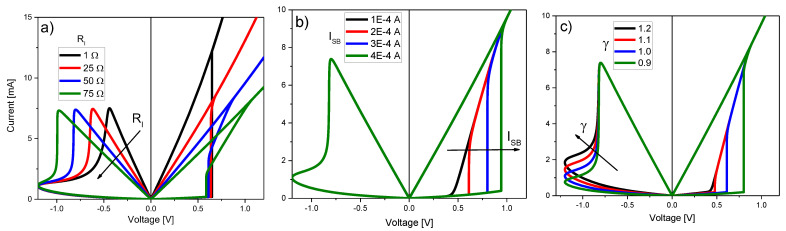
Effect of the DMM parameters on the *I-V* characteristic: (**a**) internal/external series resistance *R_I_*, (**b**) snapback triggering current *I_SB_*, and (**c**) snapforward coefficient λ.

**Figure 6:**
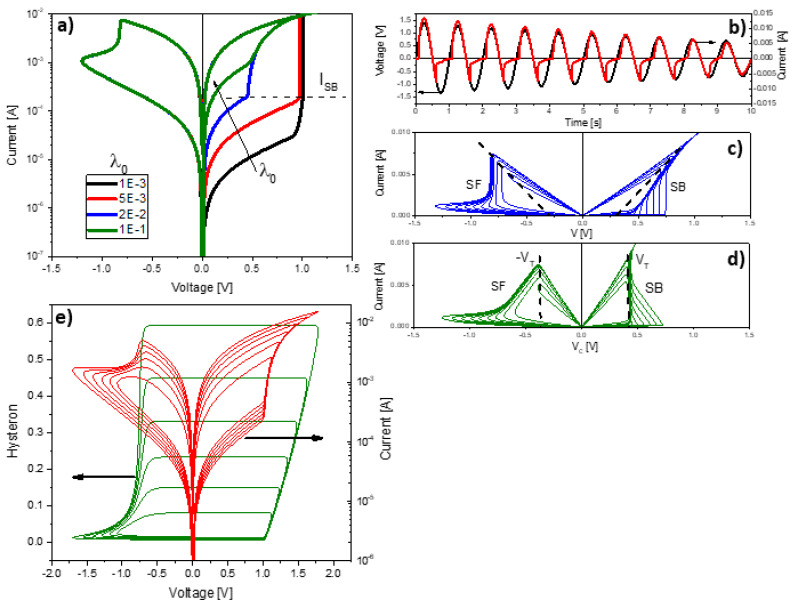
Effect of the initial memory state *λ_0_* on the *I-V* characteristic. (**a**) Effect of the initial memory state λ on the I-V characteristic. (**b**) Application of a damped sinusoidal voltage on the memdiode *I-t* characteristic, (**c**) *I-V* curve with minor loops, and (**d**) *I-V_C_* curve. *V_T_* is the transition voltage. (**e**) Simulation of intermediate states in the I-V characteristic without considering SB effect.

Figure 5 illustrates the effects of some of the model parameters on the *I-V* curve. The analysis is carried out on the second *I-V* loop, i.e., once the transient effects associated with the initial loop or forming process plays no role. As shown in Figure 5a, $R_{I}$ mainly affects the slope of the LRS *I-V* curve and the apparent RESET voltage. The small shift in the SET voltage is a consequence of the modifications that occurred in the RESET region of the first loop (not shown). Remarkable changes are also observed in the *I-V_C_* curve (see below). Figure 5b illustrates the effects of the threshold current *I_SB_*. As this parameter increases, the completion of the filament takes place at a higher voltage, thus reducing the observable effects associated with *V_T_*. No change is detected in the RESET transition since the LRS *I-V* remains unaltered. Figure 5c illustrates the effects of the SF parameter γ. The main effect on the RESET transition is the change of the triggering point of the current lobe. Since the HRS current in the RESET region is also affected by this change, γ also alters the triggering point in the SET region. It is also important to mention that the observation of the SB effect in the simulated curve strongly depends on the memory state initial condition (*λ_0_*). As shown in Figure 6a, depending on the HRS current magnitude, the SB triggering point can differ (because the same current value is reached at different voltages). Once the device reaches LRS, the RESET process becomes independent of the initial condition. Subsequent loops do not carry on information about the initial state of the device.

A remarkable property of the *I-V_C_* curve, which results from the SB transformation (*V-R·I*) of the original *I-V* curve (see Figure 6b,c), is that in addition to the current increase at a constant voltage *V_T_* occurring in the SET region, the minor *I-V_C_* loops also peak at *-V_T_* in the RESET region (see Figure 6d) for *V_R_ = −V_T_*. This has been experimentally verified in [60] and indicates that the constriction voltage or alternatively the field and not the current magnitude are responsible for triggering the RESET process. As another example of the switching dynamics that can be achieved with the DMM, Figure 6e shows the case in which the SB effect is not considered. Notice the hard threshold voltage for the SET condition. The *I-V* curve (red line) shows the intermediate current states (minor loops) for a damped sinusoidal input voltage. These states are generated by the hysteron (λ) shown in green.

### 3.2. Switching Dynamics

**Figure 7:**
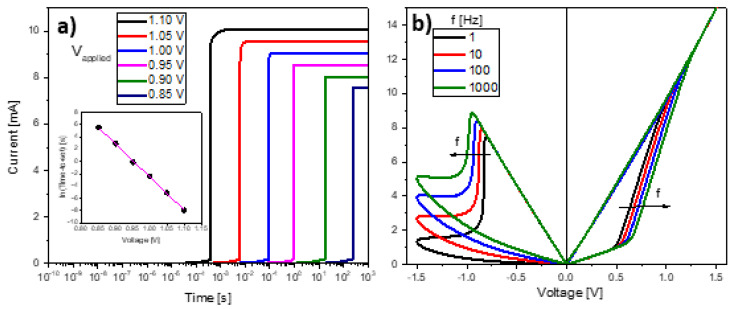
(**a**) Effect of a constant voltage input signal on the *I-t* characteristic. The inset shows the time-to-switch as a function of the applied voltage. (**b**) Effect of a sinusoidal signal with frequency *f* on the *I-t* characteristic.

As the second criterion for assessing the model behavior, the DMM switching dynamics is discussed next. It is worth emphasizing that the switching properties of the memory Equation (8) for constant and ramped voltage signals were reported in [46]. Briefly, Equation (8) complies with the expected characteristic switching times (SET and RESET) for a constant bias condition (Figure 7a):

(13) $$\tau_{S,R}\left(V\right)=\tau_{0S,R}e^{\mp V/V_{0S,R}}$$

and with the switching voltage value as a function of the applied signal ramp rate (*RR*) (Figure 7a):

(14) $$V_{S,R}=V_{S,R_{0}}ln\left(RR\right)+V_{S,R_{0}}ln\left(\frac{\tau_{S,R_{0}}}{V_{S,R_{0}}}\right)$$

where $\tau_{S,R_{0}}$ and $V_{S,R_{0}}$ are the fitting constants. As shown in Figure 7a, as the constant applied voltage increases, the maximum reachable current not only increases but also the SET switching time reduces in an exponential manner (see the inset in Figure 7a). This has been experimentally demonstrated to occur in many material systems [61]. A similar behavior is obtained for the RESET transition of the device. Although no close-form expression for a sinusoidal input is available, the phenomenology is similar to that expressed by Equation (14) for a voltage ramp. Figure 7b illustrates the effects of the signal frequency on the *I-V* curve. As the frequency increases, the SET and RESET voltages shift to higher values. This effect is consistent with Equation (14) and has been experimentally observed using ramp rates varying orders of magnitude [62]. Physically, the reason behind this behavior is the voltage-time combined action in the characteristic switching times for the ionic/vacancy hopping represented by Equations (9) and (10). The current increase observed in the RESET transition region (*V < 0*) of Figure 7b has also been observed, as it is a consequence of the increment of the current lobe triggering point [62].

**Figure 8:**
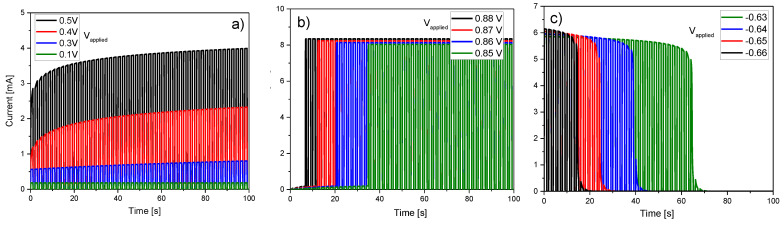
(**a**) Effect of a pulsed signal on the SET I-t characteristic, (**b**) similar to (**a**) but with a higher voltage, and (**c**) similar to (**a**) but for a RESET I-t curve.

Concerning the switching dynamics for discontinuous signals, Figure 8 illustrates the effects of a sequence of equal amplitude voltage pulses (*V_applied_* = 0.1, 0.3, 0.4, 0.5 V) and period (*T* = 1 s with duty cycle = 0.5 s) on the current magnitude. As shown in Figure 8a, for a device with an initial memory state *λ*_0_ = 0 (HRS), the current increases as function of voltage and time. This corresponds to the so-called potentiation effect in neuromorphic devices [63]. In addition, for higher voltages, as shown in Figure 8b, the current not only progressively increases but also switches to LRS after reaching the threshold condition dictated by the SB effect (pulse-induced switching). For negative voltages (see Figure 8c), the current behaves in a similar fashion. First, the current decreases monotonically but as soon as the RESET condition is met, the current exhibits an abrupt reduction. In this latter case, the device memory state starts at *λ*_0_ *=* 1 (LRS).

### 3.3. CRS Devices

Complementary Resistive Switching consists in the anti-serial combination of two memristive devices [64,65]. This is the third criterion selected for evaluating the DMM. This is an emblematic problem to demonstrate the connection capacity of the model devices. CRSs are intended to be used as selector devices in crossbar arrays with the aim of reducing the crosstalk effect [55,66]. Different behaviors are experimentally observed depending on the voltage and current window investigated including progressive and abrupt transitions [67,68,69,70,71]. Most of the RS models published to date are unable to cope with all these behaviors within a single framework.

**Figure 9:**
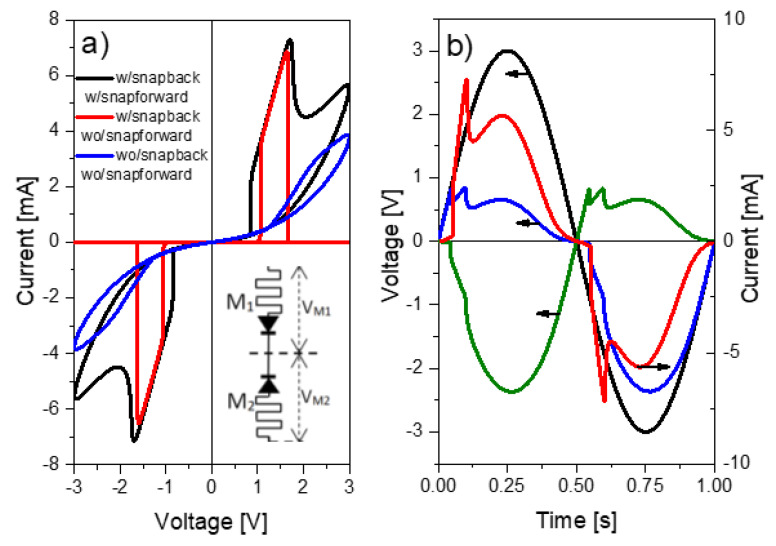
(**a**) I-V characteristic for a CRS structure w/o SB and SF. (**b**) Voltage drop distribution as a function of time.

As shown in Figure 9a, the top device (DMM1) is initially in HRS (*λ*_0_ = 0) and the bottom device (DMM2) in LRS (*λ*_0_ = 1). Figure 9 illustrates the combined action of both memdiodes in the stationary loop. The current behavior is characterized by the appearance of two bumps (transmission windows) at opposite voltages. The high current state is reached when both devices are in LRS. Remarkably, different behaviors can be achieved depending on the specific features selected in the simulation model. Figure 9a illustrates three cases of particular interest. In general, the inclusion of the SB effect yields abrupt HRS/LRS transitions, while the absence of the SB effect leads to progressive transitions. The inclusion of the SF effect with $\lambda^{\gamma }\neq 1$ in Equation (11) results in the appearance of a lobe current in the LRS/HRS transition. In order to understand the complexity of the analyzed problem, Figure 9b illustrates the potential drop distribution across each device as a function of time for the circuit shown in the inset of Figure 9a when a sinusoidal signal is applied. The figure illustrates the cases with SB and without SF effects.

## 4. Experimental Validation

**Figure 10:**
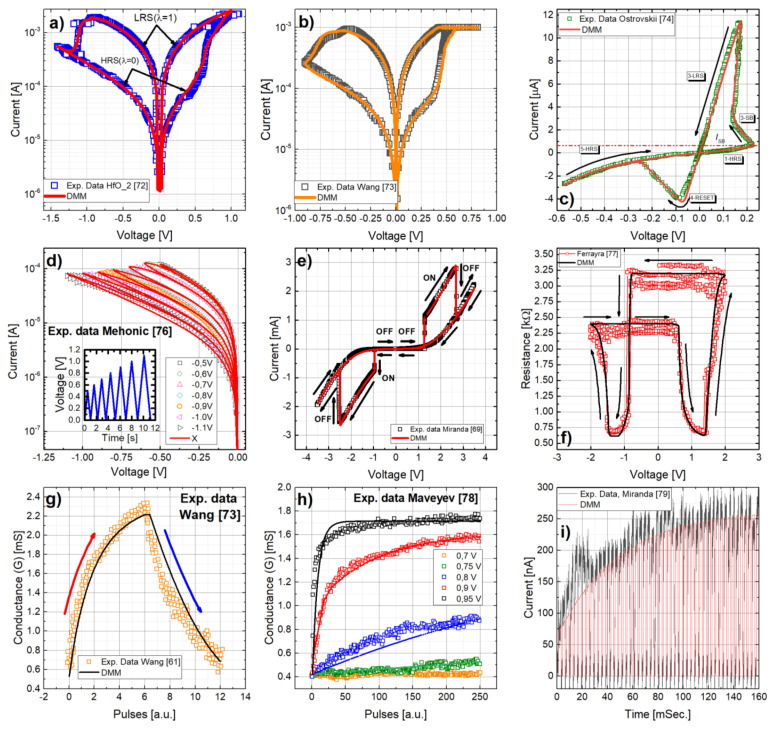
Experimental *I-V* loops of different memristor structures reported in the literature fitted with the DMM model: (**a**) Ta/HfO_2_/Pt [72], (**b**) TaO_X_ [73], and (**c**) W dopped Ge_2_Se_3_ [74,75]. As reference, the HRS and LRS curves are indicated in (**a**). Note that in (**b**) a current compliance of 1 mA was imposed to prevent permanent dielectric breakdown, which can be also represented by the DMM and SPICE. (**d**) Experimental and simulation results for the reset characteristics of SiO_X_ from UCL (data from [76]) using the QMM model. The inset shows the input signal. CRS: Experimental and simulated I-V characteristics. The arrows indicate the direction of the applied bias: (**e**) Pt/Ta_2_O_5_/Ta [69] and (**f**) Pt/Ta_2_O_4.7_/TaO_1.67_/Pt [77]. (**g**) Pulse-enabled LTP and LTD data from [73] was also used to test the suitability of the DMM to replicate the potentiation and depression behavior of memristor with multiple intermediate states. (**h**) LTP dependence on the pulses amplitude [78] can also be captured with the DMM. (**i**) The versatility of the DMM also allow fitting the current measured during the LTP of a Ag/ZnO/Pt nanowire memristive device [79].

The model discussed in the previous sub-sections was put under test by fitting experimental data extracted from different published works, both for Valence Change Memories (VCM) and Electrochemical Memories (ECM). In particular, Figure 10a,b shows the results obtained for two different VCM-type RRAM structures with HfO_2_ [72] and TaO_X_ [73] as the dielectric layer. Figure 10c presents the fitting results for a commercially available ECM device [74] comprising a W dopped Ge_2_Se_3_ active layer. In all the cases the *I-Vs* were measured at room temperature and under voltage sweeps. The experimental data were fitted using the SPICE model described in Algorithm 1 based on Equations (6) to (11) and applying driving signals as described in the corresponding references. The fitting parameters are listed in Table 1 as a reference. It should be mentioned that the DMM does not only provide a simple SPICE-compatible implementation for the resistive memory devices but also a versatile one, as it can accurately fit the *I-V* loops experimentally measured in different RRAM devices, while accounting for their particular features (as the current limitation in Figure 10b and the snapback effect in Figure 10c). Note that in Figure 10c, the *x*-axis accounts for the voltage effectively applied to the memristive device, (*V-IR*) that results after subtracting the voltage drop in the series resistance (*R_S_*, which in the experiments is reported to be 46.25 kΩ [75]).

A critical requirement for analog memristors is to account for intermediate states between the HRS and LRS regimes, which allow for a fine -tuning of the synaptic conductance in neuromorphic circuits and the storage of more than one bit per device in memory applications. This is experimentally presented in Figure 10d for a RRAM stack comprising a SiOx dielectric layer [76], which is accurately modelled by the DMM model. In order to fully represent the intermediate states from LRS to HRS altogether with the major *I-V* loops, seven successive ramped voltage pulses with increasing amplitude were considered, as shown in the inset of Figure 10d. As previously discussed, the model also allows to represent CRS devices, which describes the combination of two bipolar memristive regimes with opposite polarities in a single device (as proposed by Linn et al. [64]). Figure 10e shows the experimental and simulation results for the *I-V* characteristic observed for the Pt/Ta_2_O_5_/Ta structures considered in [69]. Note the sharp opening of the ON-state window caused by the inclusion of the SB effect. For the particular case of symmetric systems (that is, those presenting two similar metal/oxide memristive structures), the representation of the device resistance as a function of the applied voltage results in the so-called “table with legs” shape [80]. In these cases, the two interfaces of the active switching layer behave in a complementary way [81]: when one switches from LRS to HRS, the other switches inversely. Such a shape can also be described by using memdiode devices, as shown in Figure 10f, for the case of the Pt/Ta_2_O_4.7_/TaO_1.67_/Pt stack studied in [77].

Moreover, we have tested the capability of the DMM to replicate the Long-Term Potentiation (LTP) and Depression (LTD) of memristive synapses used in neuromorphic hardware. This kind of evolutionary behavior is required to achieve gradual conductance changes upon pulse applications. Figure 10g shows the LTP and LTD measurements reported in [73] for TiN/(25 nm)TaO_X_/Pt-based devices (the experimentally measured *I-V* loop is plotted in Figure 10b altogether with the corresponding fit with the DMM) by the application of 300 identical pulses of 1 V (LTP) followed by 300 identical pulses of –1.1 V (LTD). All pulses have the same width (100 ns, *t_ON_*) and they are applied every 20 msec. During the time in between pulses, a low voltage (0.1 V) pulse is applied to read the conductance (memory) state of the device. These measurement conditions were replicated in the SPICE simulations shown in Figure 10b. The simulated LTP and LTD trends are superimposed to the measurements shown in Figure 10g, showing a good fit of the experimental trends. There is also a voltage acceleration of the LTP/LTD trends, as reported for instance for the TiN/(3-nm)HfO_2_/Pt-based devices measured in [78], which can be reproduced by the DMM, as shown in Figure 10h. Finally, the versatility and capability of the DMM to reproduce the evolutionary behavior of memristive devices is shown (see Figure 10i) by also fitting the gradual current increase during LTP of Ag/ZnO/Pt-based nanowire memristors [79] by considering the same stimuli (pulses with an amplitude of 2.5 V and a duration of roughly 2 msec.). Thereby, the DMM is suitable to model the response of RRAM devices with a large number of incrementally accessible conductance states. For the sake of completeness, the fitting parameters for all the cases covered in Figure 10 are summarized in Table 1.

## 5. Conclusions

A compact behavioral model for the *I-V* characteristic of bipolar resistive switching devices was presented. The Dynamical Memdiode Model relies on the combined action of two equations, one for the electron transport based on an extension of the quantum-point contact model and a second one for the internal memory effect that represents the ion/vacancy displacements. It was shown how the snapback and snapforward effects play a fundamental role in the SET and RESET processes, respectively. The model equations were implemented in the LTSpice simulator but can be easily translated to any other specific simulation language. The model can deal with arbitrary input signals, continuous or discontinuous, derivable or not. Fine tuning of the model equations could be required for specific situations. In summary, the proposed model is simple, robust, and accurate, as required for a fast and reliable simulation involving resistive switching devices.

## Figures and Tables

**Table 1:** List of parameters used to fit the data presented in Figure 10.

Work	Material	I_min_ [A]	I_max_ [A]	α_min_ [a.u.]	α_max_ [a.u.]	R_Smin_ [Ω]	R_Smax_ [Ω]	η_SET_	η_RESET_	V_SET_ [V]	V_RESET_ [V]	I_SB_ [A]	Gam	V_T_ [V]
[72]	Ta/HfO_2_/Pt	80 µ	1.1 m	2	2.75	100	150	8	10	600 m	−575 m	300 µ	0	350 m
[73]	TaO_X_	75 µ	1.5 m	2.4	4	120	120	40	7	375 m	−130 m	1	0.05	350 m
[74]	W-Ge_2_Se_3_	500 n	50 µ	4.3	1.75	10	10	50	250	200 m	−20 m	700 n	0.35	50 m
[76]	SiO_X_	1 µ	60 µ	3	3	1k	1	20	20	395 m	−395 m	1	1	350 m
[69]	Pt/Ta_2_O_5_/Ta	3 µ	0.9 m	3	1.75	160	160	50	50	2.4	−1.35	60 µ	0.3	0
		2 µ	0.9 m	4	3	160	160	50	50	1.15	−1.05	40 µ	0.3	0
[77]	Pt/Ta_2_O_4.7_/TaO_1.67_/Pt	24.5 µ	200 µ	2	2	10	10	15	50	900 m	−670 m	30 µ	2	600 m
		17 µ	140 µ	2	2	100	100	100	50	750 m	−820 m	50 µ	3	650 m
[79]	Ag/ZnO/Pt	450 p	3.5 n	2	2	200	200	2.4	10	1	−1	1	0	1

## Data Availability

The fitting data is available from the respective reference. The DMM model fitting each dataset is available in the following Bitbucket repository: https://bitbucket.org/fernando_aguirre/uab_dmm/src/master/ (accessed on 19 January 2021).

## Supplementary Materials

The following supporting information can be downloaded at: https://www.mdpi.com/article/10.3390/mi13020330/s1, Supplementary Figure S1 “Simulation of the hysteron (top panel) and I-V characteristic (bottom panel) using a sinusoidal input “, Supplementary Figure S2 “Simulation of the forming I-V characteristic (first loop) and the stationary I-V curve (second loop) using a sinusoidal input “ and Supplementary Figure S3 “Simulation of the I-V characteristic with current compliance”.
