# Supplementary material for: SPICE Implementation of the Dynamic Memdiode Model for Bipolar Resistive Switching Devices
Source: Micromachines (Basel). 2022 Feb 19;13(2):330. doi: 10.3390/mi13020330 (PMC8874538; doi:10.3390/mi13020330)
Supplement: Supplementary file 1 [file micromachines-13-00330-s001.zip › micromachines-1586970-supplementary.pdf]

SPICE Implementation of the Dynamic Memdiode Model for Bipolar Resistive Switching Devices

Supplementary Material

Fernando Leonel Aguirre *, Jordi Suñé and Enrique Miranda *

Departament d'Enginyeria Electrònica, Universitat Autònoma de Barcelona (UAB), 08193 Barcelona, Spain; jordi.sune@uab.cat
* Correspondence: aguirref@ieee.org (F.L.A.); enrique.miranda@uab.cat (E.M.)

For the sake of completeness, and to help the reader in the use of the DMM, this Appendix illustrates three simulation exercises in LTSpice obtained with the script reported in ALGORITHM 1:

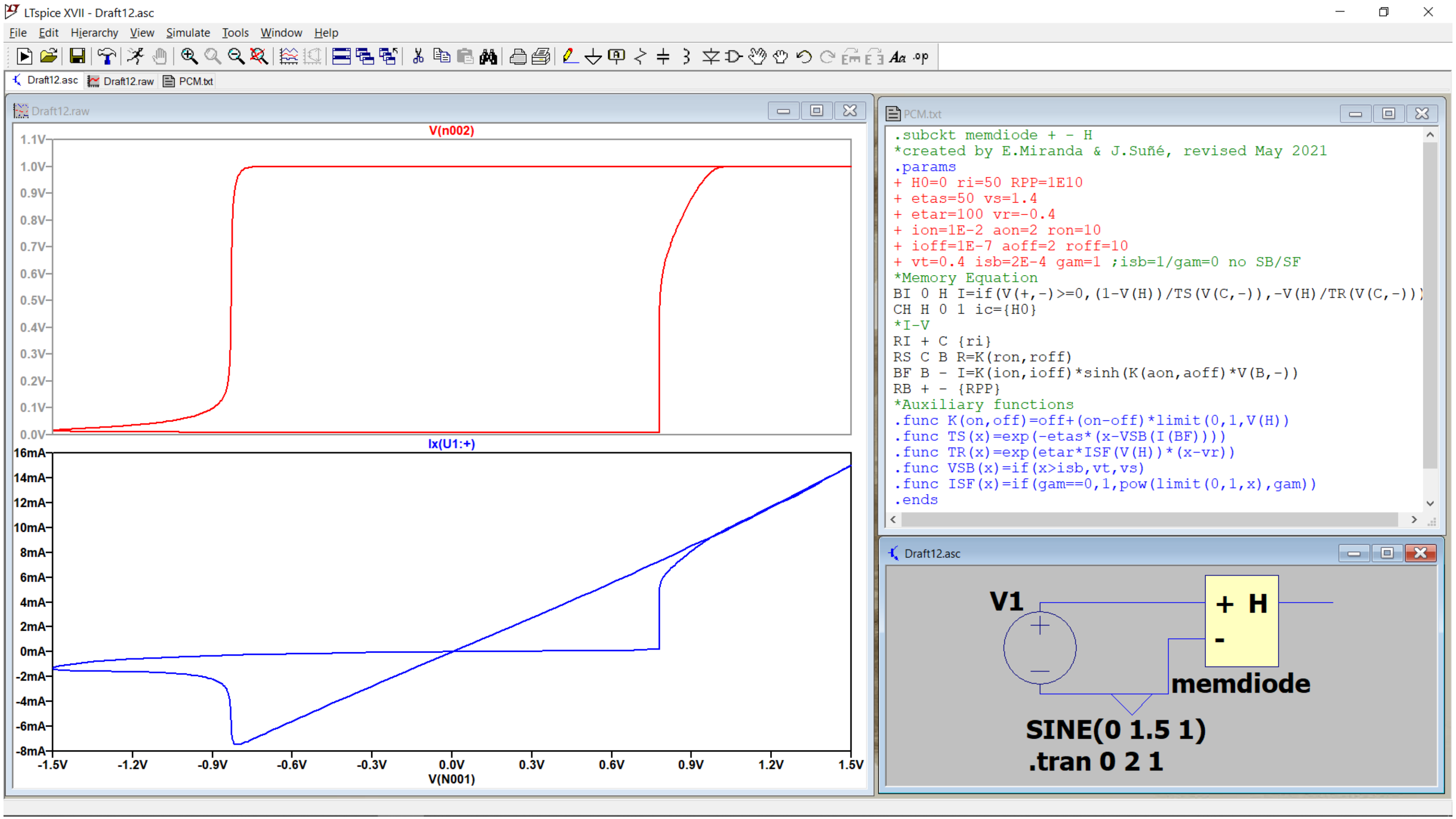

Supplementary Figure S1. Simulation of the hysteron (top panel) and I-V characteristic (bottom panel) using a sinusoidal input.

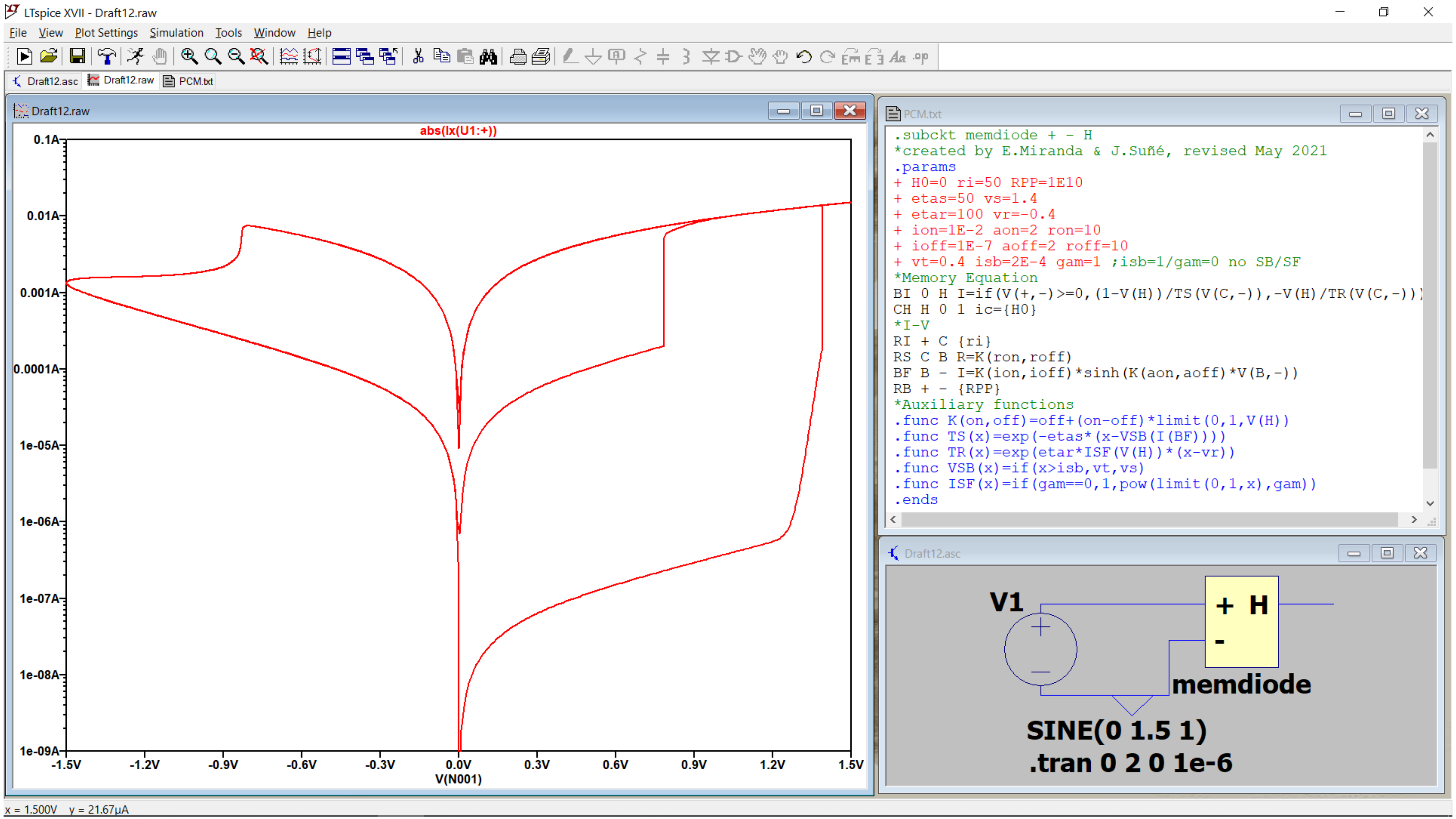

Supplementary Figure S2. Simulation of the forming I-V characteristic (first loop) and the stationary I-V curve (second loop) using a sinusoidal input.

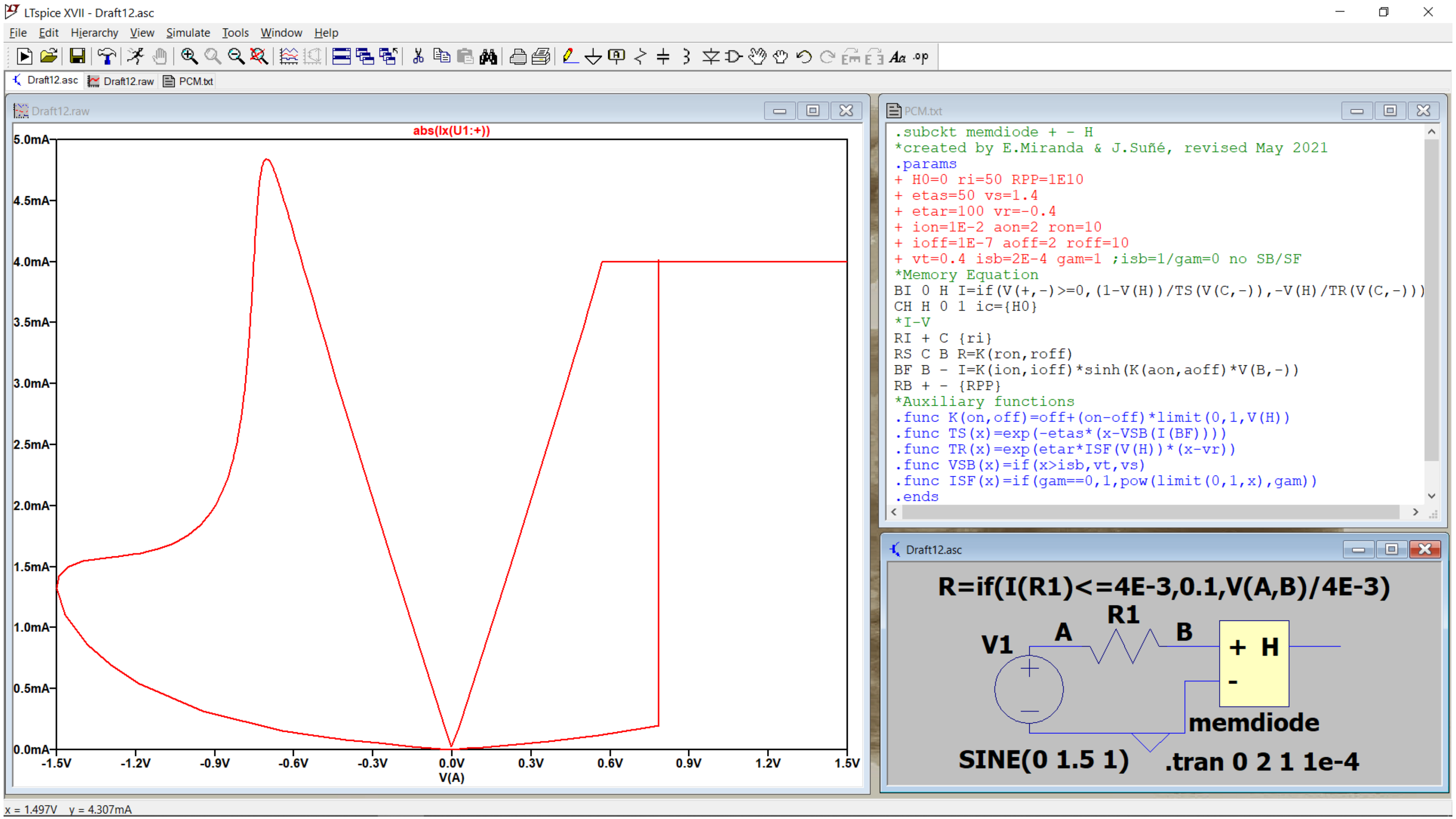

Supplementary Figure S3. Simulation of the I-V characteristic with current compliance.
